# Association between self-reported METs and other perioperative cardiorespiratory fitness assessment tools in abdominal surgery—a prospective cross-sectional correlation study

**DOI:** 10.1038/s41598-024-56887-5

**Published:** 2024-04-03

**Authors:** Szymon Czajka, Łukasz J. Krzych

**Affiliations:** 1 Department of Anaesthesiology and Intensive Care, Faculty of Medical Sciences in Katowice, Medical University of Silesia, Medyków 14, 40-772 Katowice, Poland; 2 Department of Anaesthesiology and Intensive Therapy, Silesian Centre for Heart Diseases, Zabrze, Poland; 3 Department of Acute Medicine, Faculty of Medical Sciences in Zabrze, Medical University of Silesia, Katowice, Poland

**Keywords:** Medical research, Risk factors

## Abstract

Cardiovascular complications represent a significant proportion of adverse events during the perioperative period, necessitating accurate preoperative risk assessment. This study aimed to investigate the association between well-established risk assessment tools and self-reported preoperative physical performance, quantified by metabolic equivalent (MET) equivalents, in high-risk patients scheduled for elective abdominal surgery. A prospective cross-sectional correlation study was conducted, involving 184 patients admitted to a Gastrointestinal Surgery Department. Various risk assessment tools, including the Revised Cardiac Risk Index (RCRI), Surgical Mortality Probability Model (S-MPM), American University of Beirut (AUB)-HAS2 Cardiovascular Risk Index, and Surgical Risk Calculator (NSQIP-MICA), were utilized to evaluate perioperative risk. Patients self-reported their physical performance using the MET-REPAIR questionnaire. The findings demonstrated weak or negligible correlations between the risk assessment tools and self-reported MET equivalents (Spearman’s ρ = − 0.1 to − 0.3). However, a statistically significant relationship was observed between the ability to ascend two flights of stairs and the risk assessment scores. Good correlations were identified among ASA-PS, S-MPM, NSQIP-MICA, and AUB-HAS2 scores (Spearman’s ρ = 0.3–0.8). Although risk assessment tools exhibited limited correlation with self-reported MET equivalents, simple questions regarding physical fitness, such as the ability to climb stairs, showed better associations. A comprehensive preoperative risk assessment should incorporate both objective and subjective measures to enhance accuracy. Further research with larger cohorts is needed to validate these findings and develop a comprehensive screening tool for high-risk patients undergoing elective abdominal surgery.

## Introduction

There are 310 million major surgical procedures carried out worldwide each year. It is estimated that 4–5% of patients undergoing these surgeries will die within 30 days after surgery; serious postoperative complications will affect 15% of them and 5–15% will be re-admitted to hospital within 30 days^1^. It should be assumed that due to the dynamic development of public health programmes and clinical medicine achievements, population growth and aging of societies, these statistics may increase in the coming years.

Cardiovascular complications represent a significant proportion of serious adverse events in the perioperative period. To ensure safety of the performed procedures and to minimize the risk of the aforementioned negative consequences of surgeries, the perioperative risk should be properly estimated. This risk depends majorly but not only on the patient’s condition and the type of surgery planned.

According to the recent ESC Guidelines endorsed by ESAIC, the patient-related cardiovascular risk is determined by the patient’s age, the presence or absence of cardiovascular risk factors or established cardiological condition and other comorbidities. Numerous tools for assessing perioperative cardiovascular risk have been developed. In addition to the meticulous clinical assessment of the patient, the guidelines point out the appropriateness of using scales such as the Revised Cardiac Risk Index (RCRI), the Surgical Risk Calculator (2011), the American College of Surgical Quality Improvement Program (ACS NSQIP), the Surgical Outcome Risk Tool (SORT) or the American University of Beirut (AUB)-HAS2 Cardiovascular Risk Index^2^.

Moreover, the available methods of preoperative evaluation include preoperative assessment of frailty syndrome and patient's functional capacity. However, the actual utility of these means of assessing the cardiovascular risk is being scientifically debated, and the wider use of frailty assessment scales and interview-based assessment of functional capacity has been questioned^3,4^.

Metabolic equivalent (MET) is a physiological measure that corresponds to the metabolic cost of daily activity. One metabolic equivalent (MET) is equal to resting oxygen consumption. The average value of one MET in humans is defined as 3.5 mL/kg/min. A patient's ability to perform 4 METs (or 14 mL/kg/min) has long been considered an indicator of patient fitness sufficient to safely undergo anaesthesia for non-cardiac surgery. Nevertheless, the clinical value of functional capacity assessment based on interview involving MET evaluation has been questioned as not being sufficiently objective^5,6^. It should be noted that analyses of the relationship between anaesthesia risk-assessment scales and patient-reported physical capacity expressed in MET have not yet been performed extensively. The studies published previously compared various approaches to assessing cardiovascular fitness, primarily focusing on establishing correlations between experimentally measured physical capacity and that assessed through questionnaires. Another frequently examined aspect was the relationship between preoperative physical fitness assessment and the occurrence of perioperative complications^7,8^.

In our study, we attempted to explore the relationship between the results of patients' preoperative assessment using scores and scales, widely used in anaesthesia and surgical practice to evaluate the risk of adverse cardiovascular events, and the preoperative physical performance results obtained from the MET self-assessment questionnaire in high-risk patients scheduled for at least intermediate risk elective abdominal surgery. Up to our knowledge, this is the first comprehensive assessment of the associations between several tools used for preoperative risk assessment in this specific clinical setting.

## Material and methods

### Study design and patients

**Figure 1:**
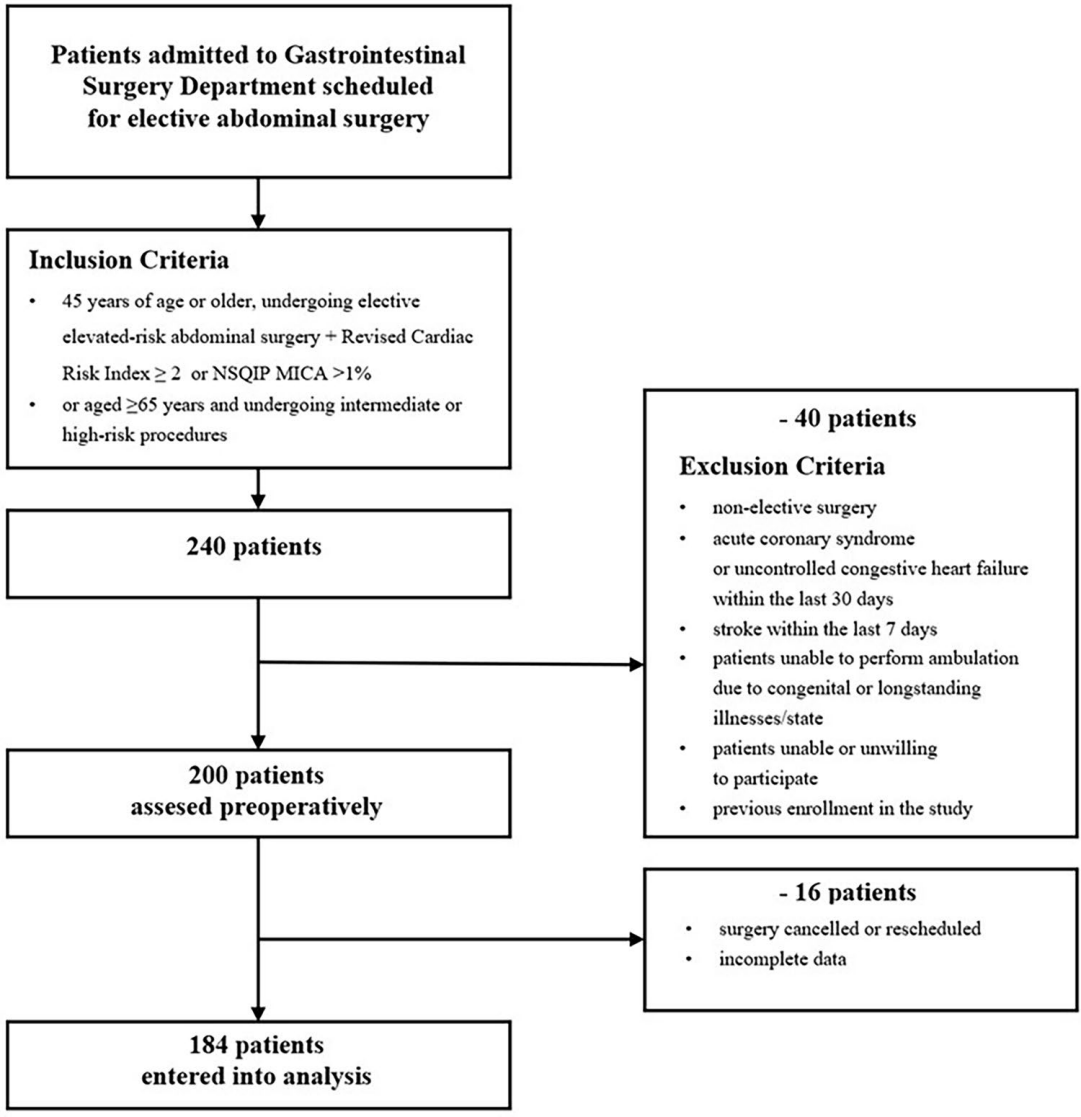
Study flow diagram.

We performed a single-centre, prospective cross-sectional correlation study focused on patients admitted to the Gastrointestinal Surgery Department of the university clinical hospital between July 2018 and December 2019. As part of the routine pre-anaesthesia consultation in the Gastrointestinal Surgery Department, attending anaesthesiologists (in training or consultant) identified patients potentially eligible for inclusion in the study. Patients scheduled for elective non-cardiac surgery aged ≥ 45 years and at increased risk of cardiovascular complications as determined by the RCRI result of ≥ 2 or National Surgical Quality Improvement Program risk calculator for Myocardial Infarction and Cardiac Arrest (NSQIP MICA) result of > 1% or aged ≥ 65 years and undergoing intermediate or high-risk surgery were suitable for inclusion. The patients who underwent surgery more than once during the study (even during separate hospital stays) were evaluated before the first procedure. All cases where the data necessary to calculate the parameters under study were missing were excluded from the analysis. The process of selection and inclusion of patients in the study is shown in the study flow chart (Fig. 1).

The study was reviewed by the Bioethics Committee of the Medical University of Silesia in Katowice and, due to its non-interventional character, it was not necessary to obtain the Committee's consent to conduct the study. (Articles 21 and 22 of the Act of 5 December 1996 on the medical profession in Poland). The Bioethics Committee stated in its decision (No. KNW/0022/KB/161/18) that the written consent of the committee was not required. The study was conducted in accordance with applicable local and international law and the principles of the Declaration of Helsinki. All patients gave their written informed consent for medical procedures and data management. The study protocol has not been published previously.

### Questionnaire, METs calculation and other collected data

**Figure 2:**
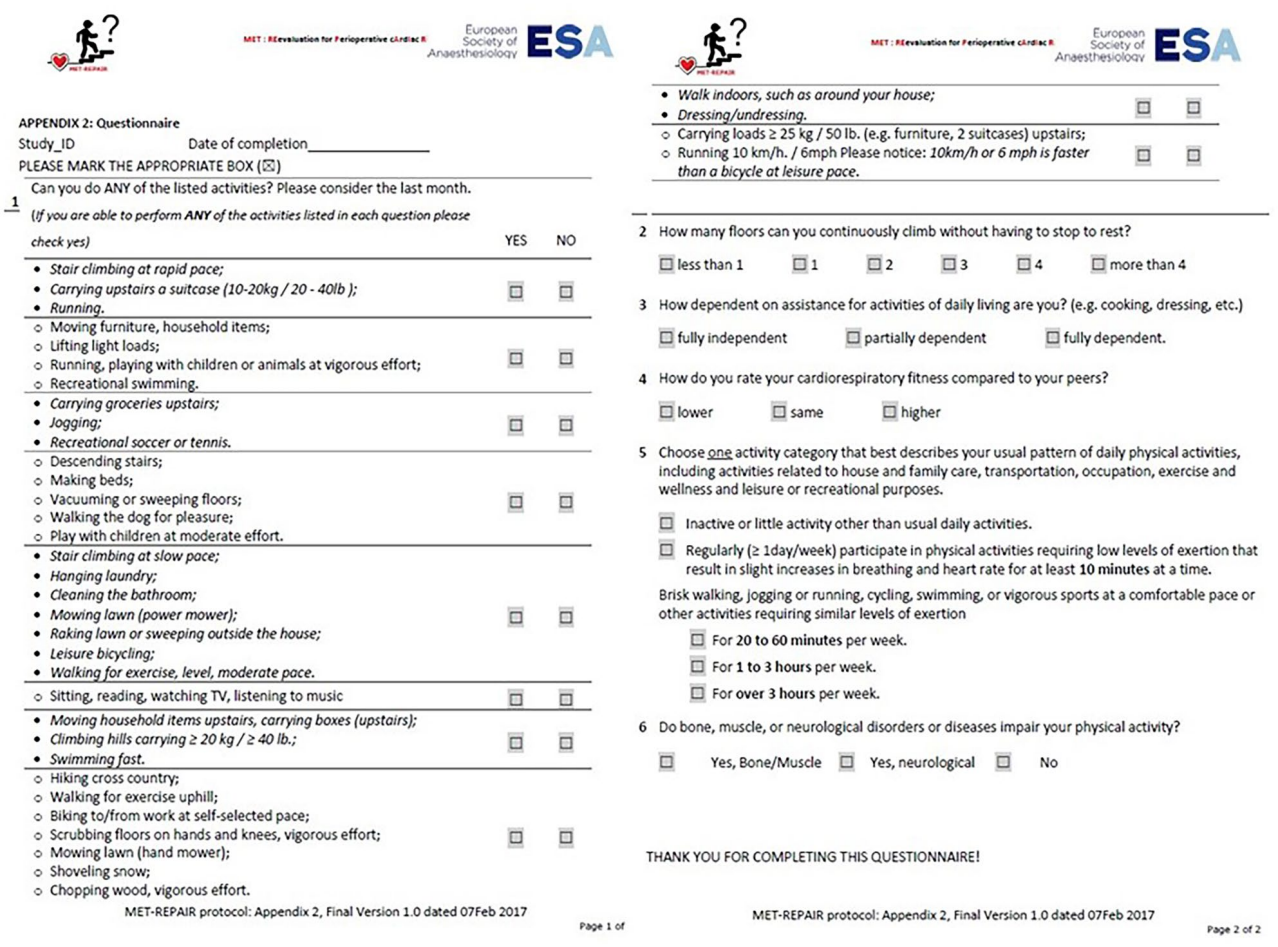
ESA Questionnaire.

Attending anaesthesiologists (in training or consultant) performed preoperative patient assessment using the validated Polish version of the questionnaire provided by the European Society of Anaesthesiology (Brussels, Belgium) during the performance of the MET-REPAIR Study (Fig. 2). The researchers were trained in the questionnaire application prior to the onset of the study. Briefly, the questionnaire consists of 2 parts, the first of which lists 10 questions for the patient's self-assessment of maximal physical performance. For each of the physical activity questions asked in this section, the patient should answer yes/no on whether they were able to perform the activity. In each question, several types of physical activity were listed to eliminate the situation where the patient did not perform a certain activity for reasons other than poor physical capacity. The questions in the first section relate to 8, 5, 7, 3, 4, 1, 7.5, 6, 2 and 8.5 METs, respectively. Two methods were used to determine self-declared METs: 'first no' and 'last yes'. For both methods, 10 questions were ordered from lowest to highest MET values (1, 2, 3, 4, 5, 6, 7, 7.5, 8, 8.5 METs). For the 'first no' method, the maximum MET was the value associated with the question preceding the first 'no' in the so ordered list of activities. The "last yes" method considered the absolute maximum number of self-reported METs, regardless of the previous answers.

Section two of the questionnaire contained four single questions: (1) number of flights of stairs that can be climbed without resting, (2) dependence on others in everyday life (3) subjective cardiorespiratory fitness in relation to the patient's peers and (4) the best characteristics of the patient's actual physical activity.

Perioperative risk was assessed using several complementary tools. These tools represent different approaches to perioperative risk assessment and differ in structure, complexity, degree of validation and implementation into clinical practice. Data available in the hospital's digital records as well as from the pre-anaesthesia anamnesis were used to calculate these parameters. Anaesthesia-related patient risk was classified with standard use of the ASA-PS classification^9^. Global procedural risk was assessed using the Surgical Mortality Probability Model (S-MPM)^10^, which was developed for non-cardiac patients and includes the patient risk (according to the ASA-PS classification), procedural risk and urgency of the procedure (emergency or non-emergency). The S-MPM predicts the risk of postoperative death in three classes: Class I—expected mortality < 0.5%, Class II—expected mortality 1.5–4% and Class III—expected mortality > 10%. It is not a scale that has entered widespread use, but with its balanced combination of patient- and procedure-related risks, it appears to be unique and its clinical utility has been previously validated^11^. The Revised Cardiac Risk Index (RCRI) was used to estimate the patient's risk of perioperative cardiac complications. This scale takes into account 6 variables; the risk for cardiac death, nonfatal myocardial infarction, and nonfatal cardiac arrest were assessed respectively with 0 predictors = 3.9%, 1 predictor = 6.0%, 2 predictors = 10.1%, ≥ 3 predictors = 15%. Another tool used was the American University of Beirut (AUB)-HAS2 Cardiovascular Risk Index (AUB-HAS-2)^12^, which considers the patient's age, preoperative haemoglobin concentration, history of cardiovascular disease, as well as the risk of vascular and urgent procedures. The risk of an adverse event defined as death, myocardial infarction, or stroke was assessed according to AUB-HAS-2 scores as follows: 0–0.3%, 1–1.6%, 2–5.6%, 3–11% and ≥ 4–17.5%. This relatively new index has already undergone external validation and is one of the tools recommended in the latest ESC guidelines. The Gupta Perioperative Myocardial Infarct or Cardiac Arrest (MICA) calculator, derived from the National Surgical Quality Improvement Program (NSQIP), was used to assess the risk of intraoperative or postoperative myocardial infarction and cardiac arrest after non-cardiac surgery^13^. This calculator is based on the type of surgery, functional status, serum creatinine level, American Society of Anesthesiologists (ASA) grade and increased age as variables. Variables used to calculate the scales and indices mentioned above are reported in Supplementary Table 1.

### Statistical analysis

Analysis was performed using MedCalc Statistical Software version 18.1 (MedCalc Software Ltd., Ostend, Belgium). Continuous variables were expressed as median and interquartile range (IQR). Qualitative variables were expressed as absolute values and/or percentages. Inter-group differences for quantitative variables were assessed using the Mann–Whitney U-test or Kruskal–Wallis test. Their distribution was verified with the Shapiro–Wilk test. The chi-square test or Fisher’s exact test were applied for qualitative variables. Odds ratios (OR) with their 95% confidence intervals (CI) were calculated, if applicable. All tests were two-tailed. We examined the correlations of self-reported physical activity (METs from the MET-REPAIR Questionnaire) and the results obtained from cardiovascular risk assessment tools using Spearman’s ρ coefficient were examined. We defined Spearman’s ρ as weak, fair, good, and excellent (< 0.4, 0.4–< 0.6, 0.6–< 0.75, and > 0.75 respectively).

## Results

A total of 240 patients were screened (Fig. 1). Of these, 40 patients did not meet eligibility criteria. In total, 200 patients were assessed before the planned surgery. In further 16 cases, the procedure was cancelled, or the data collected were incomplete. This left 184 patients with complete questionnaires and the remaining dataset analysed in the study.

Table 1 presents the study group characteristics. Basing on the “first no” assessment method described above, patients performed a median of 4 METs (IQR: 4–5). Thirty-five patients were unable to perform more than 4 METs. The majority of patients (62.5%) were classified as ASA-PS class III. The median cardiac event risk calculated using RCRI, AUB-HAS2 and NSQIP-MICA were 6 (6–10.1), 1.6 (0.3–5.6) and 0.95 (0.95–1.18), respectively. The majority of 140 patients (76.1%) had arterial hypertension and 44 (23.9%) were previously treated for coronary artery disease;137 patients (74.5%) were diagnosed with oncological illness.

**Table 1:** Study group characteristics.

General variables	Value
Male sex	87 (47.3%)
Age (years)	69 (66–75)
Height (cm)	165 (160–171)
Weight (kg)	72 (63–82.3)
BMI (kg/m^2^)	26.3 (23.4–29.2)
Obesity (BMI ≥ 30 kg/m^2^)	32 (17.4%)
History of arterial hypertension	140 (76.1%)
History of diabetes	58 (31.5%)
History of coronary artery disease	44 (23.9%)
Previous myocardial infarction	20 (10.9%)
Previous percutaneous intervention	28 (15.2%)
Previous coronary artery bypass grafting	9 (4.9%)
Peripheral artery disease	31 (16.9%)
Severe valvular stenosis or regurgitation	12 (6.5%)
History of stroke/TIA	5 (2.7%)
Smoker	
Current	27 (14.7%)
History of smoking (> 1 month ago)	20 (10.9%)
Oncological disease	137 (74.5%)
Medication	
Platelet inhibitors	44 (23.9%)
B-blockers	104 (56.5%)
Renin–angiotensin–aldosterone Inhibitors	82 (44.6%)
Calcium channel blockers	38 (20.7%)
Statins	21 (11.4%)
Diuretics	51 (27.7%)
Type of surgery	
Anorectal	6 (3.3%)
Foregut/hepatopancreatobiliary	93 (20.5%)
Gallbladder, appendix, adrenal and spleen	17 (9.2%)
Hernia (ventral, inguinal, femoral)	10 (5.4%)
Intestinal	53 (28.2%)
Other abdominal	5 (2.7%)
ASA-PS Class	
II	61 (33.2%)
III	115 (62.5%)
IV	8 (4.3%)
METs	
“First no” method	4 (4–5) 35 (19%) < 4 METs
“Last yes” method	5 (5–7) 32 (17.4%) < 4 METs
S-MPM class	
1	90 (48.9%)
2	90 (48.9%)
3	4 (2.2%)
RCRI score	
No variables = cardiac event risk 3.9%	0 (0.0%)
1 variable = cardiac event risk 6%	108 (58.7%)
2 variables = cardiac event risk 10.1%	44 (23.9%)
≥ 3 variables = cardiac event risk 15%	32 (17.4%)
(AUB)-HAS2	
0 points = cardiac event risk 0.3%	56 (30.4%)
1 point = cardiac event risk 1.6%	79 (42.9%)
2 points = cardiac event risk 5.6%	27 (14.7%)
3 points = cardiac event risk 11%	19 (10.3%)
≥ 4 points = cardiac event risk 17.5%	3 (1.6%)
NSQIP-MICA	
Cardiac risk %	0.95 (0.95–1.18)

Qualitative variables are depicted as absolute value (and percentage); quantitative variables are shown as median (and interquartile range), *BMI* Body Mass Index, *TIA* transient ischaemic attack, *ASA-PS* The American Society of Anesthesiologists Physical Status, *METs* metabolic equivalents, *RCRI* Ravised Cardiac Risk Index, *(AUB)-HAS2* American University of Beirut (AUB)-HAS2 Cardiovascular Risk Index, *NSQIP MICA* Gupta Perioperative Risk for Myocardial Infarction or Cardiac Arrest (MICA).

As reported in Table 2, the correlations between the self-reported METs versus surgical, and cardiological fitness assessment tools were weak (i.e. Spearman’s ρ < 0.4). On the other hand, a better correlation was observed between other risk assessment tools, with the highest Spearman’s ρ in the case of NSQIP and ASA-PS (ρ = 0.830).

**Table 2:**
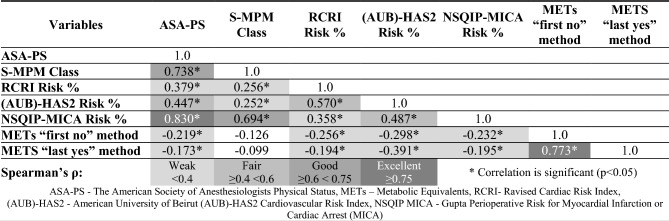
Spearman’s correlation between the studied parameters.

*Correlation is significant (p < 0.05).

*ASA-PS* The American Society of Anesthesiologists Physical Status, *METs* Metabolic Equivalents, *RCRI* Ravised Cardiac Risk Index, *(AUB)-HAS2* American University of Beirut (AUB)-HAS2 Cardiovascular Risk Index, *NSQIP MICA* Gupta Perioperative Risk for Myocardial Infarction or Cardiac Arrest (MICA).

The responses to single questions are summarised in Table 3. Responses to all analysed questions (number of flights of stairs, fitness in relation to peers, weekly physical activity, and physical dependence) were related to self-reported METs (p < 0.01). The direction and statistical significance of these relationships were confirmed in post-hoc analyses.

**Table 3:** Summary of single questions.

	N (% total)	METs “first no” method	P value	Post hoc analysis *p < 0.05
Flights of stairs			< 0.001	
< 1 flight	10 (5.4%)	2.5 (1–3)		*
1 flight	52 (28.3%)	4 (3–4)		Reference
2 flights	77 (41.8%)	4 (4–5)		*
3 flights	21 (11.4%)	4 (4–5)		*
4 flights	8 (4.3%)	5 (4–7)		*
> 4 flights	16 (8.7%)	7 (4–8.25)		*
Fitness in relations to peers			< 0.001	
Less fit	79 (42.9%)	4 (3–4)		Reference
Same fitness	92 (50%)	4 (4–5)		*
More fit	13 (7.1%)	7 (5–7.62)		*
Weekly physical activity			< 0.001	
Inactive	144 (78.3%)	4 (4–4.5)		Reference
< 20 min	22 (12%)	6 (5–7)		*
20–60 min	9 (4.9%)	7 (4.75–8.13)		*
1–3 h	6 (3.3%)	5 (5–7)		*
> 3 h	3 (1.6%)	7 (5.5–8.13)		*
Physical dependence			< 0.001	
Totally independent	163 (88.6%)	4 (4–5)		Reference
Partially dependent	20 (10.9%)	3 (3–4)		*
Totally dependent	1 (0.5%)	3		

As presented in Table 4, scores on the RCRI and S-MPM scales were not associated with the ability to climb the second flight of stairs (p > 0.05). Analyses of the other parameters studied showed a statistically significant relationship in this case (p < 0.01).

**Table 4:** Relationship between the studied risk assessment tools and patient’s ability to climb 2 flights of stairs.

Risk assessment tool	Ability to climb < 2 flights of stairs n = 62	Ability to climb ≥ 2 flights of stairs n = 122	P value
	Median (IQR)	Median (IQR)	
ASA-PS	3 (3–3)	3 (2–3)	< 0.01
S-MPM Class	2 (1–2)	1 (1–2)	> 0.05
RCRI Risk %	10.1 (6–15)	6.0 (6–10.1)	0.01
(AUB)-HAS2 Risk %	5.6 (1.6–11)	1.6 (0.3–1.6)	< 0.001
NSQIP-MICA Risk %	1.04 (0.62–1.31)	0.72 (0.21–1.12)	< 0.01
METs “first no” method	4 (3–4)	4 (4–5)	< 0.001
METs “last yes” method	4 (3–6)	7 (4–8)	< 0.001

*IQR* interquartile range, *ASA-PS* The American Society of Anesthesiologists Physical Status, *METs* Metabolic Equivalents, *RCRI* Ravised Cardiac Risk Index, *(AUB)-HAS2* American University of Beirut (AUB)-HAS2 Cardiovascular Risk Index, *NSQIP MICA* Gupta Perioperative Risk for Myocardial Infarction or Cardiac Arrest (MICA).

**Figure 3:**
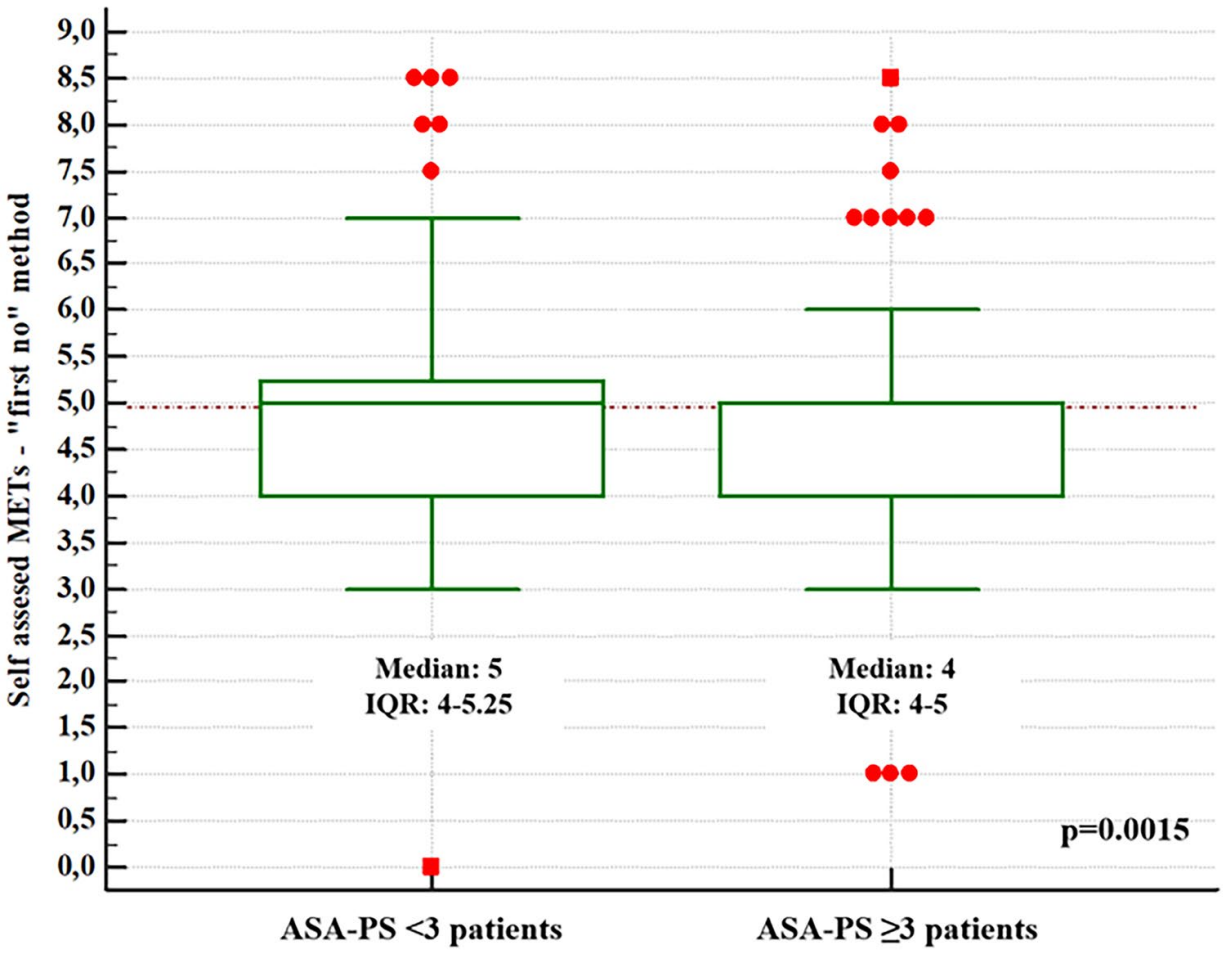
Box-and-whisker plot: self-reported MET values ("first no" method) vs the dichotomised variable ASA-PS.

**Figure 4:**
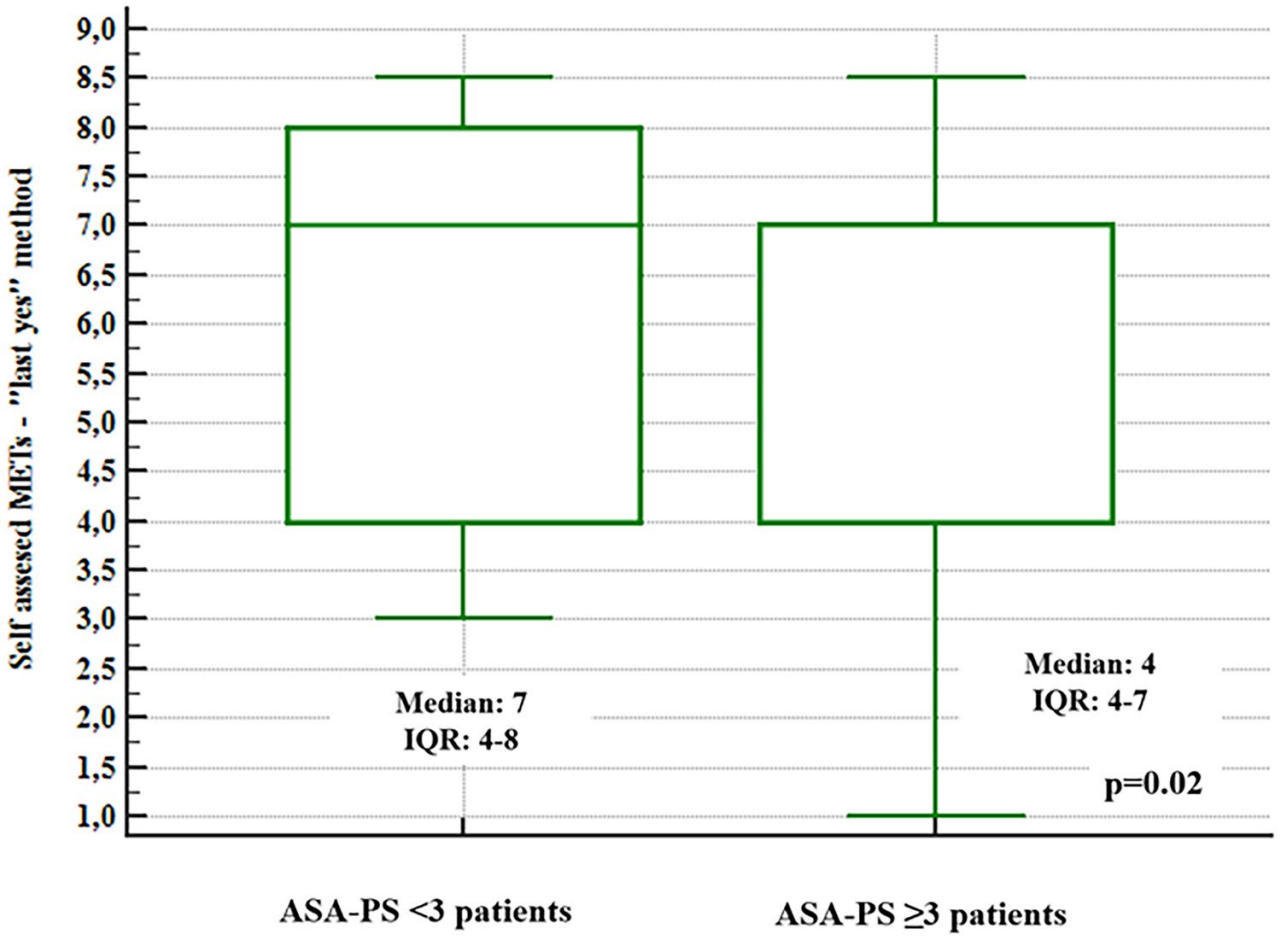
Box-and-whisker plot: self-reported MET values ("last yes" method) vs the dichotomised variable ASA-PS.

Box-and-whisker plots (Figs. 3, 4) showed the difference in self-reported MET values according to the dichotomised variable ASA-PS < III vs. ≥ III. For both methods used to determine MET values, the relationship proved statistical significance (p < 0.05).

## Discussion

The primary objective of this study was to explore the relationship between the results of patients' preoperative assessment using scores and scales, widely used in anaesthesia and surgical practice for evaluating the risk of adverse cardiovascular events, and the preoperative physical performance results obtained from the MET self-assessment questionnaire among high-risk patients scheduled for at least intermediate risk elective abdominal surgery.

To date, multiple studies have assessed the diagnostic value of MET in predicting the occurrence of postoperative complications. However, to the best of our knowledge, few have focused on the direct comparison of preoperatively assessed cardiovascular, anaesthesia and surgical risk scales with the value obtained in the preoperative assessment of functional capacity expressed in METs.

The key result of our analysis is that there is no or weak correlation between ASA, S-MPM, NSQIP-MICA, RCRI, (AUB)-HAS2 assessment tools and self-reported MET equivalents assessed preoperatively. On the other hand, the answer to a simple question about physical fitness (i.e. being able to climb 2 flights of stairs) seems to be related to the results obtained in the commonly used perioperative risk assessment scales.

The ASA Physical Status Classification System has been in use for over 60 years. During this period of time, it has undergone adaptations and modifications and its clinical usefulness and relevance is beyond doubt, but the characteristics of patients assigned to the same class can be radically different. The data obtained by Hackett et al. who have demonstrated that ASA is an independent predictor of complications in non-cardiac surgery appear to be relevant. Increases in ASA predicted significant increases in complication rates for morbidity and mortality post-operatively^14^. In the analysis by Sankar et al., the ASA-PS classification was correlated with RCRI (ρ = 0.40); moreover, it had the ability to predict in-hospital mortality (AUC 0.69) and cardiac complications (AUC 0.70)^15^. The relatively new S-MPM scale has included a component of the ASA-PS classification, surgical risk and degree of procedure urgency. It has been externally validated and proved useful in assessing perioperative risk but is not widely applied in studies^11^.

The latest ESA/ESC guidelines recommend the standard use of NSQIP, AUB-HAS2 as well as RCRI calculators in the preoperative assessment of patient's functional capacity before non-cardiac surgery^2^. All the aforementioned tools have been thoroughly validated and their usefulness in perioperative assessment in the population of patients under our study has been extensively documented. Of the tools evaluated, the NSQIP MICA is the most complex system; it is based on an online calculator and the resulting score is adapted to the specific procedure; moreover, it takes into account the largest number of variables (see Supplement). The Philippine study, comparing NSQIP and RCRI is worth mentioning. The authors have showed that the NSQIP Surgical Risk Calculator had an excellent predictive ability for MACE and was comparable with the RCRI (AUC 0.93 vs. 0.93)^16^. Despite the widespread use of RCRI and the great convenience of its application, it should be emphasised that according to some other authors, the predictive accuracy of RCRI in terms of MACE is limited and it poorly predicts the risk of postoperative death^17^. An interesting compromise between the complex but well-performing in terms of discriminative performance NSQIP and the simple-to-use but often questioned RCRI may be the AUB-HAS2 index. In the recent study, this simple-to-use risk assessment system consisting of six variables was prospectively validated in a large cohort of patients. The ROC AUC for predicting all-cause mortality, MI, or stroke was as high as 0.89^18^.

In our study, the ASA, S-MPM, as well as NSQIP and AUB-HAS2 showed mostly good correlation with each other. In particular, the NSQIP score correlated well with the ASA and S-MPM scales (ρ = 0.830 and ρ = 0.694) and fairly well with the AUB-HAS2 (ρ = 0.487). The RCRI results correlated poorly with the scores of the other risk assessment tools studied, which may be due to the mentioned drawbacks of this system described in the literature.

Higher level of cardiorespiratory fitness (CRF), which can be expressed in metabolic equivalent units (METs), is associated with lower risk of all-cause mortality, coronary heart disease and cardiovascular diseases^19^. According to the ESC/ESA 2014 guidelines, functional capacity assessment has been identified as the pivotal step in preoperative cardiac risk assessment^20^. However, there are several ways of assessing the functional capacity of patients and their results and applicability to daily clinical practice differ diametrically. METs can be determined using cardiopulmonary exercise testing (CPET), which is the gold standard, a questionnaire or simple subjective clinical assessment. According to the METS study, published in 2018 in The Lancet, the Duke Activity Status Index (DASI)^21^ questionnaire was characterised as a more precise estimate of cardiac risk than subjectively assessed functional capacity expressed in METs, and improved the risk estimation using the RCRI. In addition, performed cardiopulmonary exercise testing (CPET) did not predict 30-day mortality, postoperative myocardial infarction, or cardiac arrest. Notably, the relatively low number of primary outcomes limited the statistical power of the analysis^8^. Another study to assess the value of METs in predicting the risk of perioperative cardiovascular events was the MET -REPAIR study, the results of which were published in April 2023. In this study, the value of METs measured by a structured questionnaire (used in our study) was associated with the incidence of post-operative MACE and MACE within 30 days after surgery. However, it should be emphasised that the MET value obtained from the questionnaire did not improve the predictive values when included in the model based on clinical variables alone. The following were included in the baseline model: age, sex, ASA physical status class, estimated glomerular filtration rate, active cancer, type of surgery, diabetes mellitus, hypertension, CHF, coronary artery disease (CAD), chronic obstructive pulmonary disease, peripheral vascular disease and stroke. These are therefore variables that are commonly accepted as cardiovascular risk factors and are also used to calculate the risk assessment scales we have discussed above. The addition of functional capacity expressed in METs improved discrimination, as compared to RCRI; performance of this model was however assessed as limited. METs did not improve the discrimination when added to NSQIP MICA results^22^.

According to the validation study performed by Jaeger et al., the results obtained with the use of the MET REPAIR questionnaire incorporated in our study correlate with the METs values obtained from CPET tests. However, the interview-based methods overestimated the measured MET values^7^. Both the Jaeger et al. study and the MET REPAIR study showed an association between measured MET values and single questions about physical performance (e.g. the ability to climb 2 flights of stairs—≥ 4 METS). This is consistent with our results demonstrating both an association between the answers to the single self-assessment questions in the questionnaire and the physical fitness value dichotomised at 4 METs, as well as between ASA-PS, NSQIP, AUB-HAS2, RCRI scores and climbing the 2nd flight of stairs. In the study by Lurati Buse et al. assessing high-risk cardiovascular patients undergoing non-cardiac surgery, self-reported functional capacity of less than two flights of stairs was independently associated with major adverse cardiac events and mortality from any cause at 30 days and 1 year^23^. Furthermore, the latest European guidelines recommend the method of assessing physical capacity based on simple question-based assessment to determine whether the patient is able to perform an activity ≥ 4METs^2^. Considering all the ways of risk assessment discussed above, the use of such a simple yet validated method seems clinically useful. It is noteworthy that practising physicians are looking for scales that are simple to use on a daily basis, e.g. when consulting before surgery, and that do not consume time or require the input of many variables^24^. On the other hand, however, there is no universal tool for assessing perioperative risk; the patient`s assessment before surgery should be multifactorial, based on the thorough medical history, and the tools we discussed should not be overlooked, but should complement each other once they are matched to a particular patient.

A noteworthy strength of our study is its prospective nature. The study used the validated questionnaire, and the parameters assessed included those that would be difficult to obtain from a retrospective analysis of medical histories.

The most important limitation of this study is the limited size of our cohort; therefore, the results may be distorted by insufficient sample size, and the study may be underpowered with a lack of generalizability. It is noteworthy that patients eligible for extensive surgical procedures are mostly initially in better physical shape than the general population and may be selected already during initial surgical consultation. Moreover, our study was designed to provide a comprehensive assessment of fitness among those undergoing general surgery. As a result, we observed a high level of heterogeneity in surgical procedures; planned surgeries varied in their level of complexity and degree of surgical risk. Finally, despite the special attention given to the reliable assessment of physical fitness, observer bias as well as frequent overestimation of fitness by the respondents themselves should be mentioned. One must be cautious about uncritically applying the 2022 ESC guidelines to practice because of the potential shortcomings and their consequences discussed in the literature with regard to preoperative cardiovascular risk assessment^25^.

## Conclusions

The correlation between the results of ASA-PS, S-MPM, NSQIP-MICA, RCRI, (AUB)-HAS2 assessment tools, and self-reported MET equivalents is suboptimal. Therefore, reliable complex preoperative risk assessment of post-operative adverse events among high-risk patients scheduled for at least intermediate risk elective abdominal surgery is difficult. It seems there is still no single tool recommended for screening in this specific cohort. Both objective and subjective methods of assessment should be incorporated to increase the accuracy of estimation.

### Supplementary Information

Supplementary Information.

## Data Availability

The datasets used and/or analysed during the current study available from the corresponding author on reasonable request.
