# Supplementary material for: Association between self-reported METs and other perioperative cardiorespiratory fitness assessment tools in abdominal surgery—a prospective cross-sectional correlation study
Source: Sci Rep. 2024 Apr 3;14:7826. doi: 10.1038/s41598-024-56887-5 (PMC10991501; doi:10.1038/s41598-024-56887-5)
Supplement: Supplementary file 1 — Supplementary Information. [file 41598_2024_56887_MOESM1_ESM.docx]

**Supplementary Table 1. - Risk assessment Tools**

| Risk assessment tool | Interpretation |  |  |
| --- | --- | --- | --- |
| ASA-PS Class |  |  |  |
| - I: normal healthy patient - II: mild systemic disease - III: severe systemic disease - IV: severe systemic disease that is a constant threat to life (i.e., patient could die acutely without intervention) - V: moribund, not expected to survive without surgery   According to the guidelines published online:  <https://www.asahq.org/standards-and-guidelines/statement-on-asa-physical-status-classification-system> | | |  |
| S-MPM Class |  |  |  |
| - ASA physical status: I=0 pts, II=2 pts , III=4 pts, IV=5 pts, V=6 pts - Procedure risk: Low risk = 0 pts, Intermediate risk=1 pt, High risk=2 pts - Emergency: Nonemergent= 0 pts, Emergency surgery=1 pt | - Class I 0–4 pts mortality: *<*0.50% - Class II 5–6 pts mortality: 1.5%–4.0% - Class III 7–9pts mortality: *>*10% |  |  |
| RCRI Score |  |  |  |
| - Elevated-risk surgery :Intraperitoneal; intrathoracic; suprainguinal vascular =1 pt - History of ischemic heart disease =1 pt - History of congestive heart failure =1 pt - History of cerebrovascular disease =1 pt - Pre-operative treatment with insulin =1 pt - Pre-operative creatinine >2 mg/dL / 176.8 µmol/L =1 pt | Risk of major cardiac event:   - 0 pts = 3.9% (2.8-5.4%) - 1 pt = 6.0% (4.9-7.4%) - 2 pts = 10.1% (8.1-12.6%) - ≥3 pts = 15% (11.1-20.0%) |  |  |
| (AUB)-HAS2 |  |  |  |
| - Age, years ≥75 = 1 pt - Haemoglobin, g/dL <12 g/dL = 1 pt - History of heart disease = 1 pt - Angina or dyspnoea = 1 pt - Vascular surgery = 1 pt - Emergency surgery = 1 pt | cardiac event risk - death, myocardial infarction, or stroke   - 0 pts = 0.3% - 1 pt = 1.6% - 2 pts = 5.6% - 3 pts = 11% - ≥4 pts = 17.5% |  |  |
| NSQIP-MICA |  |  |  |
| Each value of the variable is assigned a different coefficient:   - Age (years) - Functional status : Independent, Partially dependent, Totally dependent - ASA class - Creatinine: Normal (≤1.5 mg/dL, 133 µmol/L), Elevated (>1.5 mg/dL, 133 µmol/L) - Type of procedure | Cardiac risk % = e^x^ / (1 + e^x^)  x = −5.25 + sum of the coefficients of the selected variable |  |  |
| ASA-PS - The American Society of Anesthesiologists Physical Status, METs – Metabolic Equivalents, RCRI- Revised Cardiac Risk Index , (AUB)-HAS2 - American University of Beirut (AUB)-HAS2 Cardiovascular Risk Index, NSQIP MICA - Gupta Perioperative Risk for Myocardial Infarction or Cardiac Arrest (MICA) | | | |
